# Some Remarks on Diagnosis and Treatment of Venereal Ulcers

**Published:** 1903-11

**Authors:** W. B. Emery

**Affiliations:** Atlanta, Ga.


### SOME REMARKS ON DIAGNOSIS AND TREATMENT OF VENEREAL ULCERS.

By W. B. EMERY, M.D.,
ATLANTA, GA.

There are none of the ills which flesh is heir to more common to the man of to-day than some form of disease of the genito-urinary organs, and decidedly none which gives him more genuine misery. What is more pitiable than the boy with his first case of gonorrhea, or the elderly man with that continual dribble which follows the neglected inflammatory conditions of his youth. Nor are there many conditions more distressing than a patient possessed with a venereal ulcer, regardless of its nature.

It is not my intention to enumerate the various degrees and details of the morbid conditions we find in these organs, nor would time or inclination permit me to go into the diagnosis or state the treatment we find in vogue for the various pathological conditions that exist and persist in and about these organs at the present time, but there is one class of cases which I wish to speak of briefly, and especially as to diagnosis and treatment.

The term venereal ulcer, which we apply to an erosion of the skin and mucous membrane of the genitals, is of an infectious nature, and may or may not extend to the deeper tissues.

For the sake of convenience we classify as follows:

- I. Simple or Traumatic

  - Abrasion,

  - Tear,

  - Chafe,

  - Fissure.

- II. Herpetic Ulcer.

- III. Ulcerated Balanitis.

- IV. Chancroid

  - Follicular,

  - Ecthymatous,

  - Ulcer Elevatum,

  - Serpiginous,

  - Phagedenic.

- V. Chancre.

  - The chancrous erosion,

  - The silvery spot,

  - The dry papule or patch,

  - The umbilicated papule or nodule, also called follicular chancre,

  - The purple necrotic nodule,

  - The ecthymatous chancre.

- VI. Malignant Epithelioma.

These classes will about cover all the forms we come in contact with. They may be found clean cut and distinct or modified, for it is quite often we see a mixed infection. Now as to diagnosis. To the average layman the least abrasion, scratch, tear or herpetic sore is looked upon with the greatest alarm. His mind at once fills with all the stories he has heard of infectious venereal disease. It is exceedingly pleasant for us to relieve the mind of these patients by being able to assure them there is nothing serious and with a little care—that is simple cleanliness—they need not anticipate trouble, but at the same time warn them should they not give this care the condition may become grave.

The simple ulcer (I mean by this the tear, abrasion, etc.) forms a ready avenue for serious infection. It may take on the form of the more serious types. Except in cases of simple ulcer our diagnosis should be guarded until the sore has assumed its typical shape.

Herpes progenitalis as a rule is readily diagnosed because of its acute inflammatory condition, the antecedent pain and irritation; also the fact that the patient has had similar attacks before.

Little vesicles are formed on the glans or inner surface of prepuce. This is the usual location, though they are sometimes found on the integument of prepuce, penis or pubic region.

These vesicles are filled with a serous fluid. They sometimes dry up, but frequently break down, thus forming an ulcer. We frequently see them during this stage for the first time. It is the previous history then that serves the main guide to diagnosis.

Ulcerated balanitis is quite easy of diagnosis. It is usually associated with elongated prepuce. The ulcers are generally large and quite superficial. The base is smooth and shining. The edges are not undermined as in chancroid, and there is no induration as in chancre.

To diagnose a chancroid one must be familiar with the five different types of the ulcer; also with all the varieties of venereal ulcers, for there are few which do not simulate the chancroid at some stage of their progress. The fact that chancroid occurs so frequently is more or less an aid to us. Some authors state that this ulcer forms about 80 per cent. of all venereal sores. In my experience, both in private practice and Dr. W. S. Goldsmith's Venereal Clinic, Atlanta College of Physicians and Surgeons, the per cent. is at least 80.

The ecthymatous chancroid is found on the dry portions of integument of genitals. It starts as a little red spot. Pus then forms beneath; the top then dries to a crust and beneath is a typical chancroid.

The elevated ulcer is merely where the tissues beneath the sore are edematous. Should the ulcer show a marked tendency to extend rapidly it is termed serpiginous. This lesion generally begins as a chancroidal bubo, which if left unchecked may extend over the groins and in some cases a portion of the abdomen. Should phagedena attack the chancroid it is termed phagedenic. We generally find this condition in alcoholics or persons poorly nourished. The sides and base of ulcer are gangrenous. Tissue is destroyed rapidly. The patient experiences most of the constitutional symptoms of infection. Has a rather high temperature and corresponding pulse, rigors or even a chill.

It is most important to be able to diagnose a chancre. More important than any lesion about the genitals, except perhaps malignant disease.

The chancrous erosion is most common form. It is first noticed as a small spot of excoriation—red in color. The surface is shining and free from granulations.

The silvery spot is generally situated on the glans near the meatus. It is very small and silvery in color, gradually increases in size and after a time is replaced by a shining surface which is typical. The dry patch begins as a dry, dull red spot. Is remarkably free of secretion of any character.

The unbilicated papule is a rare form. Begins as pinkish elevation with central depression. It increases in size and takes on red color.

The purple necrotic nodule is also rare. It usually occurs on the glans and about the coronal sulcus. Begins as a small dark red spot, which later on becomes purplish. This may either subside or go on to necrosis.

Ecthymatous chancre is simply a chancre with dark brown crust.

The crust is evidently the dried pus thrown off by the ulcer. All of these forms I have mentioned finally develop into superficial erosion with smooth red shining floor and profuse serous secretion. They are all situated upon and surrounded by a circumscribed mass of induration.

I desire to say a few words as to the plan I use in differentiating chancre from chancroid. In most cases the lesions are typical and easy of diagnosis, but there are some where mixed infection exists and it is utterly impossible to say whether or not the sore is the initial lesion of syphilis. In these cases I try to gain the confidence of patient and, if I can manage him, make him wait for the secondary signs to appear before beginning treatment. If necessary I resort to a placebo. I hardly think we are justified in starting the patient on a course of treatment which is to last two or more years when there is the least doubt as to diagnosis.

I have heard of what is called the therapeutic test, which is—If a patient can take more than the ordinary doses of mercury without purging or any ill effect he has the disease in question. This plan I consider unsatisfactory and apt to lead on the wrong trail.

The best plan and the safest is to wait, even the ninety days if necessary. I am satisfied there are many who are informed they have syphilis when they have not. It is far best to be slow and exceedingly careful when there is the least shadow of doubt. It is better the patient should think you a poor diagnostician than his whole life be blighted with the erroneous idea he has had the much-dreaded disease.

About the only malignant ulcer we have is epithelioma. This generally begins as a small venereal wart which becomes excoriated, ulcerated and then indurated. It occurs most frequently on the glans and prepuce and after middle age. It sometimes grows from the seat of a chancre. At times there are cauliflower-like growths located at and within the seat of the ulcer.

There is nothing much to be said about the treatment of what I have termed simple ulcers. Cleanliness and protection are the first and only principles to follow. Should there be slight infection I generally use some mild antiseptic wash, such as sodium chloride, borolyptol, boracic acid or potassium permanganate solution. After bathing, use powder composed of bismuth subnitrate and calomel, equal parts.

For herpes or ulcerated balanitis I always insist upon the patient being circumcised, thus removing the cause in a large majority of cases. When the elongated prepuce is removed, then some simple remedies as stated above will generally lead to a rapid disappearance of the ulcers. Occasionally they will persist, due to the tenderness of the integument and the uncleanly habits of patient. In these cases I advise that the parts be rubbed in alcohol and witch hazel which has both cleansing and hardening effect.

The chancroid is a lesion greatly abused by some surgeons. The tendency is to cauterize. Chemical cauterization is the treatment to be used in many cases, but there are some which would do much better without it and others too extensive.

The chemicals I use are carbolic and nitric acid. First swab the surface of ulcer with pure carbolic for the anesthetic effect, then apply thoroughly the nitric with cotton swab. A 40 per cent. formalin solution has been suggested. Some claim to have used it with success, but I have never tried it. After applying the nitric acid thoroughly I use a moist dressing which consists of absorbent cotton saturated with bichloride solution 1 to 10000. This serves to protect the ulcer and absorb the secretion; whereas a powder serves as a mechanical irritant. The only time I use a powder is when ulcer is very large, irritable and painful. I then employ a formula which consists of orthoform 1 part, bismuth subnitrate 3 parts. This secures almost perfect local anesthesia for twenty-four hours. I have discarded all ointments of every description except mercurial, and this only in a certain class of cases.

It unquestionably checks phagedena and stimulates resolution. In many of these cases potassium iodide or protoiodide of mercury are of aid to rapid recovery of patient. I frequently give as large doses of the above drugs as can be taken comfortably.

Most types of chancre need no treatment whatever except a bath of bichloride 1 to 5000 once or twice daily, and protected with a moist dressing consisting of absorbent cotton saturated with bichloride solution.

It may be necessary in a few cases to cauterize the ulcer when there is a tendency to break down rapidly. In these cases I use carbolic and nitric acid as in chancroid.

When the sore is on the prepuce and circumcision will remove it, I always advise that operation. Should it be situated within a prepuce that is unable to retract, I advise the dorsal or bilateral incision, the ulceration controlled and subsequent circumcision.

An idea has been advanced that should the initial lesion of syphilis be removed at the time of its first appearance it will mitigate the disease. This I do not believe, as there is infection of the system before the chancre appears.

In the treatment of epithelioma about the only course to be considered is the total excision of the diseased part. When the disease has not extended farther than slight ulceration of the indurated papule, it may be necessary only to remove the involved area and cauterize the wound with caustic potash. When the disease has fairly developed the only recourse is amputation of the organ carried wide of the affected area. At the same time the inguinal glans should be removed whether they be enlarged or not.

REFERENCES.—J. R. Hayden's book on Venereal Diseases. White and Martin.

